# Hollow Carbon Nanorod Confined Single Atom Rh for Direct Formic Acid Electrooxidation

**DOI:** 10.1002/advs.202205299

**Published:** 2022-11-11

**Authors:** Yezhou Hu, Changsheng Chen, Tao Shen, Xuyun Guo, Chen Yang, Deli Wang, Ye Zhu

**Affiliations:** ^1^ Department of Applied Physics The Hong Kong Polytechnic University Kowloon Hong Kong P. R. China; ^2^ Key Laboratory of Material Chemistry for Energy Conversion and Storage (Huazhong University of Science and Technology) Ministry of Education Hubei Key Laboratory of Material Chemistry and Service Failure School of Chemistry and Chemical Engineering Huazhong University of Science and Technology Wuhan 430074 P. R. China

**Keywords:** coordination‐template method, formic acid oxidation reaction, hollow porous structure, single‐atom catalysts

## Abstract

Nearly theoretical 100% atomic utilization (supposing each atom could serve as independent sites to play a role in catalyz) of single‐atom catalysts (SACs) makes it highly promising for various applications. However, for most SACs, single‐atom sites are trapped in a solid carbon matrix, which makes the inner parts hardly available for reaction. Herein, a hollow N‐doped carbon confined single‐atom Rh (Rh‐SACs/HNCR) is developed via a coordination‐template method. Both aberration‐corrected scanning transmission electron microscopy and energy dispersive X‐ray spectroscopy mapping confirm the uniform distribution of Rh single atoms. Owning to the unique hollow structure and effective carbon confinement, excessive conversion from pyridinic/pyrrolic N to graphic N is hindered. As a proof of concept, Rh‐SACs/HNCR exhibits superior activity, stability, selectivity, and anti‐poisoning capability in formic acid oxidation reaction compared with the counterpart Rh/C, Pd/C, and Pt/C catalysts. This work provides a powerful strategy for synthesizing hollow carbon confined single‐atom catalysts apply in various energy‐related systems.

## Graphical abstract

N‐doped hollow carbon nanorod confined single atom Rh (Rh‐SACs/HNCR) is prepared through a coordination‐template method. Hollow carbon nanorods not only confine single atom Rh but also maximize the exposure of inner active sites. Benefiting from the elimination of unwanted ensembles, CO poisoning is greatly avoided and preferred direct dehydrogenation dominates in Rh‐SACs/HNCR.

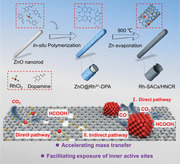

## 1 Introduction

Direct formic acid fuel cells (DFAFCs) show superiority in high energy density, portability, storage, and safety over hydrogen and other liquid fuel cells (e.g., methanol fuel cells, etc.).^[^ ^1^ ^]^ Currently, the most widely used catalysts in the anode are still Pt and Pd‐based materials.^[^ ^2^ ^]^ However, their activities still do not match their costs, and poisoning can easily happen during formic acid oxidation, greatly hindering their further applications. While alloying^[^ ^1^, ^3^ ^]^ and morphology tailoring,^[^ ^4^ ^]^ etc. can improve the formic acid oxidation reaction (FAOR) activity and stability, CO poisoning still cannot be avoided due to the ensemble effect (that larger ensembles may favor the CO adsorption, thus making unwanted dehydration pathway dominated). To eliminate the unwanted large ensembles sites, constructing isolated metal sites could be an ideal strategy.^[^ ^1d^ ^]^

Single‐atom catalysts (SACs), as the next‐generation catalysts, have drawn numerous attention due to the maximal atom utilization, unsaturated coordination environment and strong metal–support interaction.^[^ ^5^ ^]^ These exceptional features make SACs widely used in various applications, such as wastewater treatment,^[^ ^6^ ^]^ organic synthesis,^[^ ^7^ ^]^ and energy conversion,^[^ ^5^, ^8^ ^]^ etc. Based on these characteristics, tremendous efforts have been taken to design highly efficient SACs. For example, through wrapping the MoS_2_ with single Fe atom layer, the obtained MoS_2_@Fe‐N‐C exhibits enhanced electrocatalytic performance.^[^ ^8b^ ^]^ However, due to the dramatically increased surface energy with minimized metal size, the single atoms become unstable and prefer to aggregate, especially in high temperature synthesis or hash working environment. N‐doped carbon materials have long been used as the ideal support to anchor metal atoms by forming strong metal–N bonds.^[^ ^5^, ^9^ ^]^ Besides, their high conductivity, low cost and structural tunability also endow SACs with excellent catalytic performance. Among the widely used carbon supports for single atoms, hollow porous carbon materials have been regarded as one of the best due to their accelerated mass transfer capability,^[^ ^10^ ^]^ as well as the exposure of inner active sites, which is normally over‐shielded in solid counterparts (Figure S1, Supporting Information).

In this work, we developed a hollow N‐doped carbon nanorod confined single atom Rh (Rh‐SACs/HNCR) through a coordination‐template method. Dopamine (DPA) served as both the carbon precursor and chelating agent to coordinate with Rh^3+^. ZnO nanorod was chosen as a self‐sacrificed template to assist the construction of a special hollow nanorod structure. The isolated Rh single atoms confined in carbon nanorods were confirmed by aberration‐corrected scanning transmission electron microscopy (AC‐STEM) and energy dispersive X‐ray spectroscopy (EDS) mapping. As expected from its unique structure, the Rh‐SACs/HNCR exhibited superior FAOR activity, stability and selectivity compared with commonly used Rh/C, Pt/C, and Pd/C catalysts. Using the similar approach, single atom Rh confined in hollow spheres can also be acquired, demonstrating the universality of the present strategy.

## 2 Results and Discussion

### 2.1 Morphology and Structure Characterizations

**Figure 1:**
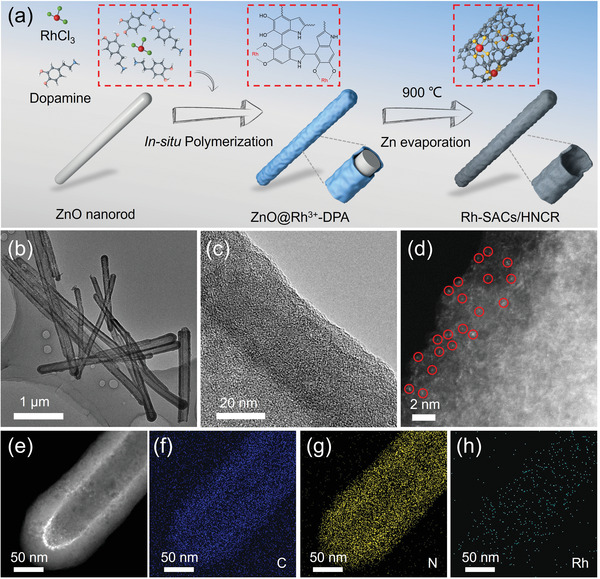
a) Schematic illustration of Rh‐SACs/HNCR. b) TEM, c) high‐resolution TEM, d) AC HAADF‐STEM, e) HAADF‐STEM images, and f–h) EDS maps of Rh‐SACs/HNCR.

The preparation of Rh‐SACs/HNCR is illustrated in **Figure** **1**a. Briefly, ZnO nanorods were synthesized as the sacrificial templates. The in situ polymerization of dopamine with the presence of RhCl_3_ leads to the formation of ZnO nanorods covered with Rh^3+^‐DPA coating layer (denoted as ZnO@Rh^3+^‐DPA). The successful coating is confirmed by the X‐ray powder diffraction (XRD) (Figure S2, Supporting Information), as indicated by a board peak at 23° along with the reduced ZnO signal. After high temperature treatment at 900 °C for evaporation of ZnO and carbonization of Rh‐containing polydopamine precursor, the Rh‐SACs/HNCR can be obtained without aggregated Rh nanoparticles (Figure S3a–f, Supporting Information). The Rh‐SACs/HNCR successfully maintains the initial nanorod morphology of ZnO template without obvious collapse. The evaporation of ZnO also gives rise to the hollow structure (Figure 1b), which is beneficial for exposing more active sites inside the carbon layer and accelerating the mass transfer process. The magnified TEM image of the carbon layer (Figure 1c) demonstrates the amorphous structure rather than the graphitized one, the latter of which normally forms after high temperature treatment. This amorphous structure ensures the strong anchoring of Rh single atoms on the carbon layer without aggregation.^[^ ^11^ ^]^ The aberration‐corrected high‐angle annular dark field (HAADF)‐STEM image (Figure 1d) indicates the atomically dispersed Rh atom on the carbon layer. EDS maps (Figure 1e–h) further confirm the uniform elemental distribution of Rh single atoms and the formation of the N‐doped carbon nanorod. The Rh metal loading in Rh‐SACs/HNCR is determined to be 0.99 wt% by ICP‐OES. Moreover, when the heating temperature is raised to 1000 °C (denoted as Rh‐SACs/HNCR‐1000), the nanorod morphology can be well‐maintained but accompanied by a rougher surface (Figure S4a, Supporting Information). Meanwhile, the total N content of Rh‐SACs/HNCR‐1000 decreased significantly (Table S1, Supporting Information). Besides, due to the loss of the thermodynamically unstable N species, some Rh‐SACs aggregated into nanoparticles with some Rh SAC remaining (Figure S4a–c, Supporting Information). The formation of Rh nanoparticles after 1000 °C heat treatment is further confirmed by electron energy loss spectroscopy mapping (EELS) (Figure S4d–h, Supporting Information).

**Figure 2:**
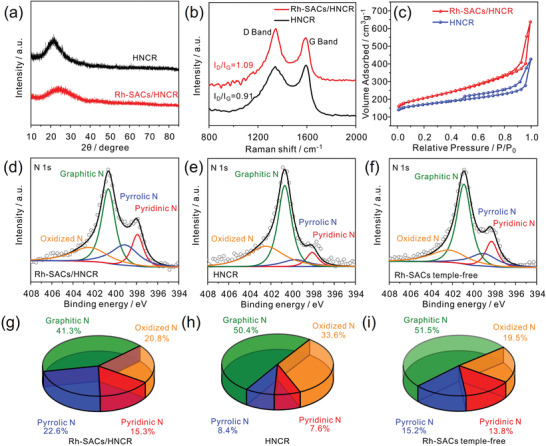
a) XRD patterns, b) Raman spectra, and c) N_2_‐adsorption/desorption isotherms of HNCR and Rh‐SACs/HNCR. High‐resolution N 1s XPS spectra and corresponding N species percentage composition of d,g) Rh‐SACs/HNCR, e,h) HCNR, and f,i) Rh‐SACs template‐free.

The structure of Rh‐SACs/HNCR is characterized using XRD (**Figure** **2**a). No diffraction peaks assigned to Rh nanoparticles can be observed but only a broad carbon characteristic peak at ≈25°, suggesting the successful formation of Rh single atoms confined in carbon nanorods. Besides, compared with the XRD pattern of HNCR, the carbon characteristic peak in Rh‐SACs/HNCR is much broader, demonstrating the much more amorphous structure of carbon nanorods with the introduction of Rh single atoms. This could be caused by the chelation effect of dopamine with Rh^3+^ during in situ polymerization.^[^ ^12^ ^]^ Thus, in subsequent high temperature treatment, the formed Rh—N/O bonds inhibit the favorable graphitization process. Indeed, Raman analysis (Figure 2b) reveals a much higher *I*_D_/*I*_G_ value (1.09) in Rh‐SACs/HNCR compared with HNCR (0.91), indicating that the doped Rh single atoms in the carbon matrix greatly increases the defect density in Rh‐SACs/HNCR despite the high temperature treatment. Besides, the impact of doped Rh single atoms on specific surface area (SSA) and pore size distribution (PSD) of hollow carbon nanorod was also evaluated by N_2_ adsorption–desorption isothermals (Figure 2c and Figure S5, Supporting Information). Both Rh‐SACs/HNCR and HNCR exhibit type IV hysteresis, suggesting their similar hierarchically porous structures. The calculated SSA of Rh‐SACs/HNCR is 680.5 m^3^ g^−1^, which is much higher than that of HNCR (549.5 m^3^ g^−1^). This result demonstrates that doped Rh single atoms can prevent the collapse of pore structure during high temperature, thus greatly maintaining the high SSA of Rh‐SACs/HNCR.

One drawback of high temperature treatment is that it tends to cause dramatic loss of N in the carbon matrix, and unstable N moieties such as pyridinic N and pyrrolic N are more likely to be converted to thermally stable graphic N.^[^ ^13^ ^]^ This problem can be largely mitigated by the preferential bonding between the Rh single atoms and pyrrolic N/pyridinic N,^[^ ^14^ ^]^ which may help to preserve them against graphic N. To investigate the influence of confined Rh atomic sites on the existing form of N species, the N 1s X‐ray photoelectron spectroscopy (XPS) of Rh‐SACs/HNCR and HNCR are compared (Figure 2d,e). The N 1s spectra could be divided into four peaks, namely pyridinic N (397.9 eV), pyrrolic N (399.1 eV), graphitic N (400.7 eV), and oxidized N (402.4 eV).^[^ ^15^ ^]^ The Rh‐SACs/HNCR shows a much higher ratio of (pyridinic N + pyrrolic N)/total N (37.9%, Figure 2g) than HNCR (16.0%, Figure 2h), suggesting that the anchoring effect between Rh single atoms and dopamine could be strong enough to inhibit the conversion from pyrrolic/pyridinic N to graphitic N. Besides, the total N content in Rh‐SACs/HNCR (2.65%) is also higher than that in HNCR (1.66%), demonstrating the critical role of single atom Rh in fixing N species at high temperature. The influence of hollow structure on the total N content and distribution of N species is also explored. The solid Rh‐SACs shows lower total N content (2.23%) and the ratio of (pyridinic N + pyrrolic N)/total N (28.9%) than Rh‐SACs/HNCR (Figure 2f,i), suggesting the N loss during high temperature treatment is possibly inhibited by the buffering effect of the hollow structure or the excess Zn evaporation.^[^ ^16^ ^]^ When annealing temperature is higher, the N 1s spectrum of Rh‐SACs/HNCR‐1000 shows significantly dropped content of N species and the ratio of (pyridinic N + pyrrolic N)/total N relative to Rh‐SACs/HNCR (Figure S6, Supporting Information), this is consistent with the previously mentioned issue that thermally unstable pyridinic N and pyrrolic N are easy to be decomposed, thus leading to the loss of total N contents. The decomposition of pyridinic N and pyrrolic N would inevitably lead to the conversion of Rh SAC to Rh nanoparticles, which has been verified through the TEM image of Rh‐SACs/HNCR‐1000 shown in Figure S4 (Supporting Information).

### 2.2 Electrochemical Formic Acid Oxidation Reaction Performance

**Figure 3:**
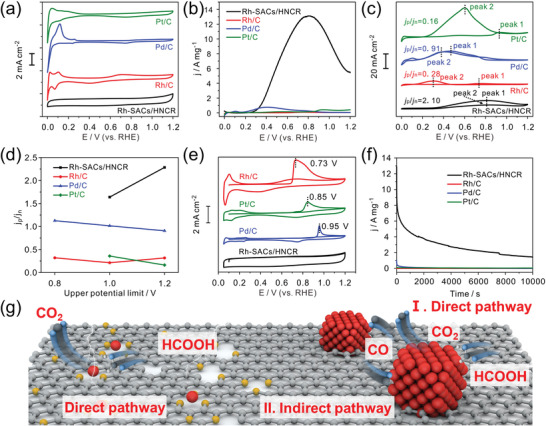
a) CVs of Rh‐SACs/HNCR, Rh/C, Pd/C, and Pt/C. b,c) Mass‐normalized activities, d) CVs for FAOR. e) CO stripping curves and f) chronoamperometry tests at 0.5 V. g) Schematic diagram of the possible FAOR pathway for Rh‐SACs/HNCR and Rh/C.

To demonstrate the benefit of the achieved unique structure with isolated Rh atoms confined in hollow carbon nanorods, the Rh‐SACs/HNCR was used as an electrocatalyst for FAOR. The electrochemical behavior of Rh‐SACs/HNCR was first investigated and compared with typical control catalysts (Rh/C, Pd/C, and Pt/C, see Figure S7, Supporting Information for their XRD patterns and TEM images) by collecting the cyclic voltammetry (CV) profiles in 0.5 m H_2_SO_4_ (**Figure** **3**a). Unlike Pt/C, Pd/C, and Rh/C, Rh‐SACs/HNCR exhibited completely different electrochemical behavior with no signal of H desorption/adsorption and metal oxidation/reduction but only a near rectangular CV profile. This is because the adsorption of hydrogen tends to happen on successive metal sites rather than an isolated one.^[^ ^1d^ ^]^ In Figure 3b, the mass normalized LSV curves demonstrate that an orders‐of‐magnitude increase in mass normalized FAOR activity could be achieved for Rh‐SACs/HNCR (13.1 A mg^−1^) compared with Rh/C (0.09 A mg^−1^), Pd/C (0.75 A mg^−1^), and Pt/C (0.42 A mg^−1^), indicating the superior FAOR activity in Rh‐SACs/HNCR. The superior FAOR performance can also be verified from the comparison with previously reported values shown in Table S2 (Supporting Information). To exclude the possible contribution of N‐doped carbon support to FAOR, the FAOR performance of HNCR is evaluated (Figure S8, Supporting Information). No formic oxidation current could be detected, verifying the role of Rh‐SACs as active sites. The electrochemical behavior of Rh‐SACs/HNCR‐1000 was also compared with Rh‐SACs/HNCR. Figure S9a (Supporting Information) shows the CV profiles, and the additional H adsorption/desorption peaks could be observed, which can be attributed to the aggregation of Rh atoms into nanoparticles, providing successive metal sites for H adsorption/desorption. Accordingly, the Rh‐SACs/HNCR‐1000 exhibits a slightly higher CO stripping volume along with more negative CO stripping potential (Figure S9b, Supporting Information). The normalized FAOR performance further shows a dramatic decrease with the partial conversion from single atoms to nanoparticles (Figure S9c,d, Supporting Information). These results show the different electrochemical behavior between single atoms and nanoparticles, and the FAOR on nanoparticles tend to be accompanied with CO poisoning.

The ratio of anodic peak current density (*j*_p_, peak 1) to cathodic current density (*j*_n_, peak 2) could be used as a FAOR selectivity descriptor.^[^ ^1b^ ^]^ These values for Rh‐SACs/HNCR, Rh/C, Pd/C, and Pt/C were calculated based on the CV curves in 0.5 m H_2_SO_4_ + 0.5 m HCOOH, respectively (Figure 3c). The Rh‐SACs/HNCR shows the highest ratio of *j*_p_/*j*_n_ (2.10), followed by Pd/C (0.91), Rh/C (0.28), and Pt/C (0.16). This indicates the direct dehydrogenation pathway in Rh‐SACs/HNCR. By controlling the upper potential and calculating the corresponding *j*_p_/*j*_n_ values, the difference in electrochemical behaviors for FAOR in these catalysts becomes more pronounced (Figure 3d, Figure S10, Supporting Information). When the upper potential changes from 0.8 to 1.2 V, Rh‐SACs/HNCR exhibits a relatively high *j*_p_/*j*_n_ value, and the *j*_p_ value is always higher than the *j*_n_ value (Figure S10a, Supporting Information), suggesting no adsorbed CO (CO_ad_) accumulation and the dominated direct dehydrogenation pathway for FAOR. For Rh/C, Pd/C, and Pt/C, they all exhibit decreased *j*_p_/*j*_n_ values with increasing upper potential (Figure S10b–d, Supporting Information). This is because the CO_ad_ occupies the active sites at low potential and inhibits the direct dehydrogenation pathway first. Once the CO_ad_ is oxidized at higher potential, the occupied active sites on ensembles would be released, then the direct dehydrogenation reaction becomes favorable along with the increased cathodic current density. Notably, for Rh‐SACs/HNCR and Rh/C, further increased *j*_p_/*j*_n_ values, as the upper potential is shifted to 1.2 V, are possibly due to the irreversible oxidation of Rh at higher potential. The *j*_p_/*j*_n_ value with the upper potential of 0.8 V for Pt/C is not given here, because the 0.8 V potential is not positive enough to remove CO_ad_ from Pt ensembles. This can also be verified from the negligible FAOR current density with an upper potential of 0.8 V (Figure S10c, Supporting Information). For Rh‐SACs/HNCR, the value at 0.8 V is not mentioned, because 0.8 V is so close to the FAOR peak potential, large error can be caused when calculating the *j*_p_/*j*_n_ value at 0.8 V. These results suggest that superior FAOR selectivity could be achieved by constructing isolated Rh active sites in Rh‐SACs/HNCR. Its electrochemical behavior could be completely different from the nanoparticle electrocatalysts, largely due to the lack of successive metal sites.

To further validate the role of CO_ad_ (as the inhibitor of active sites) and investigate its influence on the selectivity of catalysts, CO stripping curves of Rh‐SACs/HNCR, Rh/C, Pd/C, and Pt/C were collected to investigate the CO resistance of catalysts (Figure 3e). For Rh‐SACs/HCNR, no CO oxidation current could be detected, possibly due to the weakened CO adsorption on Rh‐SACs/HNCR. Obvious CO oxidation current with different oxidation peak potentials could be observed for Rh/C (0.73 V), Pt/C (0.85 V), and Pd/C (0.95 V), indicating that CO oxidation is more likely to happen to Rh/C, followed by Pt/C and Pd/C. For Rh/C and Pt/C, the CO oxidation peak potentials are close to their CO poisoning peak potentials (Peak 1 observed in Figure 3c), confirming that the indirect dehydration pathway dominates in Rh/C and Pt/C. Despite that CO adsorbed on Pd/C is more difficult to be removed until 0.95 V (Figure 3e, blue line), nearly no CO poisoning peak can be observed in its CV for FAOR (Figure 3c, blue line), indicating the direct dehydrogenation pathway is more favorable than indirect dehydration pathway in Pd/C for FAOR. Based on the above analysis, we attribute the superior FAOR selectivity of Rh‐SACs/HNCR to its insensitivity toward CO, as isolated Rh atomic sites would be no more available for the CO_ad_‐formation involved dehydration pathway but be more favorable for the direct dehydrogenation pathway.^[^ ^5d^ ^]^ The model for different FAOR pathways between the Rh‐SACs/HNCR and control catalysts is shown in Figure 3g. The FAOR stability of Rh‐SACs/HNCR was also evaluated through chronoamperometric tests at 0.5 V for 10 000 s (Figure 3f), with the FAOR current of Rh‐SACs/HCNR always orders of magnitude higher than that of the control catalysts after.

### 2.3 Structure Stability Characterization After Stability Test

**Figure 4:**
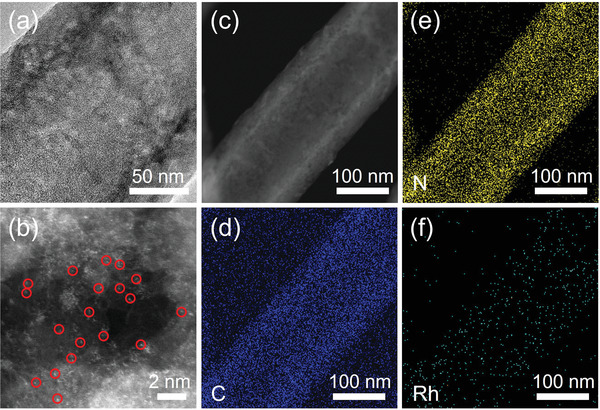
a) High‐resolution TEM, b) AC STEM, c) HAADF‐STEM and corresponding EDS maps of Rh‐SACs/HNCR after stability test.

The structure stability was later evaluated by TEM after stability tests (**Figure** **4**a). It can be observed that the hollow structure is well maintained, and Rh single atomic sites are largely preserved without the formation of Rh nanoparticles (Figure 4b). The HAADF‐STEM image and corresponding EDS maps (Figure 4c–f) further confirm the uniform distribution of Rh atomic sites confined in the hollow N‐doped carbon nanorod. These results suggest the superior structure stability of Rh‐SACs/HNCR during FAOR.

### 2.4 Universal Synthesis of Rh SACs Confined in Multi‐Dimensional Carbon Support

**Figure 5:**
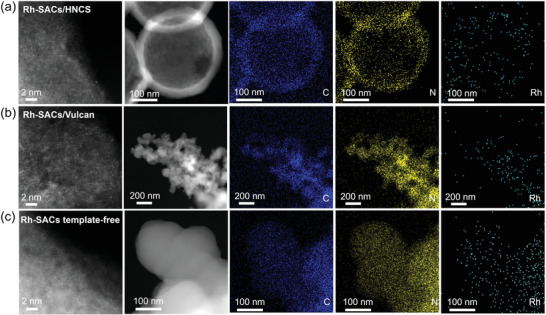
HAADF‐STEM images and corresponding EDS maps of a) Rh‐SACs/HNCS, b) Rh‐SACs/Vulcan, and c) Rh‐SACs template‐free.

The present synthesis method can also be used to prepare Rh SACs confined in multi‐dimensional carbon supports, including Rh SACs confined in 3D hollow N‐doped carbon spheres (Rh‐SACs/HNCS, **Figure** **5**a), 0D solid carbon black (Rh‐SACs/Vulcan, Figure 5b), and also without using template (Rh‐SACs template‐free, Figure 5c). The isolated Rh single atoms in these catalysts were confirmed by corresponding AC HAADF‐STEM images. No nanoparticles can be observed for these catalysts and corresponding EDS maps suggest the uniform distribution of Rh single atoms in various carbon supports and the formation of the N‐doped carbon matrices. The above results demonstrate the present synthesis as a general strategy to obtain Rh single atoms confined in the various multi‐dimensional carbon matrices.

## 3 Conclusion

In conclusion, hollow N‐doped carbon nanorod confined single atom Rh has been successfully obtained via a coordination‐template method. Benefiting from the easy removal of ZnO at high temperature, no post‐treatment is required. Besides, the unique hollow structure ensures the maintenance of total N content during high temperature treatment due to the possible buffering effect. The induced Rh atoms not only function as active sites but also hinder the excess graphitization process of carbon. This helps maintain the defect‐rich state of the carbon matrix. As a proof of concept, the prepared Rh‐SACs/HNCR exhibits superior FAOR activity, stability, selectivity, and CO anti‐poisoning capability, which can be attributed to the isolated Rh atom sites, unique hollow structure, carbon confinement, and accelerated mass transfer process. At last, this work also demonstrates a promising strategy for designing confined single‐atom catalysts for various applications.

## Conflict of Interest

The authors declare no conflict of interest.

## Supporting information

Supporting Information

## Data Availability

The data that support the findings of this study are available from the corresponding author upon reasonable request.
