# Supplementary material for: Hollow Carbon Nanorod Confined Single Atom Rh for Direct Formic Acid Electrooxidation
Source: Adv Sci (Weinh). 2022 Nov 11;9(36):2205299. doi: 10.1002/advs.202205299 (PMC9799016; doi:10.1002/advs.202205299)
Supplement: Supplementary file 1 — Supporting Information [file ADVS-9-2205299-s001.pdf]

Hollow carbon nanorod confined single atom Rh for direct formic acid electrooxidation

Yezhou Hu, Changsheng Chen, Tao Shen, Xuyun Guo, Chen Yang, Deli Wang, Ye Zhu**

Y. Hu, C. Chen, X. Guo, C. Yang, Y. Zhu
Department of Applied Physics
The Hong Kong Polytechnic University
Hong Kong, P. R. China
E-mail: yezhu@polyu.edu.hk

T. Shen, D. Wang
Key Laboratory of Material Chemistry for Energy Conversion and Storage (Huazhong University of Science and Technology), Ministry of Education, Hubei Key Laboratory of Material Chemistry and Service Failure
School of Chemistry and Chemical Engineering
Huazhong University of Science and Technology
Wuhan 430074, P. R. China
E-mail: wangdl81125@hust.edu.cn

Material synthesis

Synthesis of ZnO nanorods

Uniform 1D ZnO nanorods were first synthesized through a one-step hydrothermal method. Briefly, 200 mg of $ZnCl_2$, 20 g of Na_2CO_3 and 40 mL of water were mixed and under vigorous stirring for 30 mins. The well mixed compound was then transferred to a 50 mL of Teflon-lined stainless-steel autoclaves and heated at 140 ^oC for 12 h. After reaction, the while solid was washed with water and ethanol several times to remove the residual alkaline substance for later use.

Synthesis of Rh-SACs/HNCR

100 mg of ZnO nanorods were dispersed in 50 mL of tris-solution containing 0.46 mg of Rh. 200 mg of dopamine hydrochloride was then added to above solution to start the polymerization reaction. After vigorous stirring for 24 h, the Rh^{3+}-dopamine coated ZnO nanorods (ZnO@Rh^{3+}-DPA) can be achieved. To obtain carbon nanorod confined single-atom (Rh Rh-SACs/HNCR), the ZnO@Rh^{+3}-DPA was heated at 900 ^oC for 2h under Ar atmosphere with ramping rate of 5 ^oC/min.

Synthesis of Rh-SACs/HNCR-1000

Similar to the synthesis of Rh-SACs/HNCR, the Rh-SACs/HNCR-1000 could be obtained with the heating temperature of 1000 ^oC.

Synthesis of HNCR

The hollow N-doped carbon nanorod (HNCR) was achieved similar to the synthesis of Rh-SACs/HNCR but without the addition of Rh precursor.

Physical characterizations

XRD patterns were collected on a Rigaku X-ray diffractometer (SmartLab 9 kW) with Cu- Kα radiation (λ=0.1541 nm). Raman spectra were collected using a Witec alpha300 R Confocal Raman system equipped with 532 nm laser. XPS spectra were acquired on X-ray photoelectron spectrometer (K-alpha, Thermo Scientific) with a monochromatic Al Kα X-ray source (hυ=1486.6 eV). SEM images were acquired using Nova NanoSEM 450 scanning electron microscope. TEM and STEM were performed using JEOL JEM-2100F TEM/STEM operated at 200 kV. Electron energy-loss spectroscopy (EELS, by Gatan Enfina) mapping was carried out under 200 kV accelerating voltage. Aberration-corrected STEM characterizations were performed on a Thermo Scientific Spectra 300 microscope equipped with a X-FEG/UltiMono electron source, a CEOS SCORR fifth-order probe corrector, a CEOS CETCORPLUS image corrector and four-quadrant Super-X windowless silicon-drift EDS detectors. It was operated at 300 kV with a 24.4 mrad convergence semi-angle. The Rh metal content in Rh-SACs/HNCR is determined by ICP-OES on Agilent 720ES.

Electrochemical characterizations

All the electrochemical measurements were performed at CHI 660E electrochemical workstation at room temperature using three-electrodes system. Reverse hydrogen electrode (RHE) and carbon rod were chosen as reference electrode and counter electrode, respectively. The catalytic ink was prepared by dispersing 5 mg of catalysts into 1 mL of 0.1% Nafion/isopropanol solution. The glassy carbon electrode (GC, 5 mm in diameter) deposited with 3 μL ink was used as working electrode. The cyclic

voltammetry (CV) curves were collected in N_2-saturated 0.5 M H_2SO_4 solution from 0 to 1.2 V with scan rate of 50 mV/s. The Formic acid oxidation currents were collected in an N_2-saturated 0.5 M H_2SO_4+0.5 M HCOOH solution from 0 to 1.2 V with scan rate of 50 mV/s. For CO stripping test, after the working electrode was held at 0.1 V for 30 min and subsequent removal of residual CO by purging N_2 for another 30 min, the CO stripping curves were collected. Chronoamperometry (CA) tests were performed at 0.5 V in an N_2-saturated 0.5M H_2SO_4+0.5 M HCOOH solution for 10,000 s.

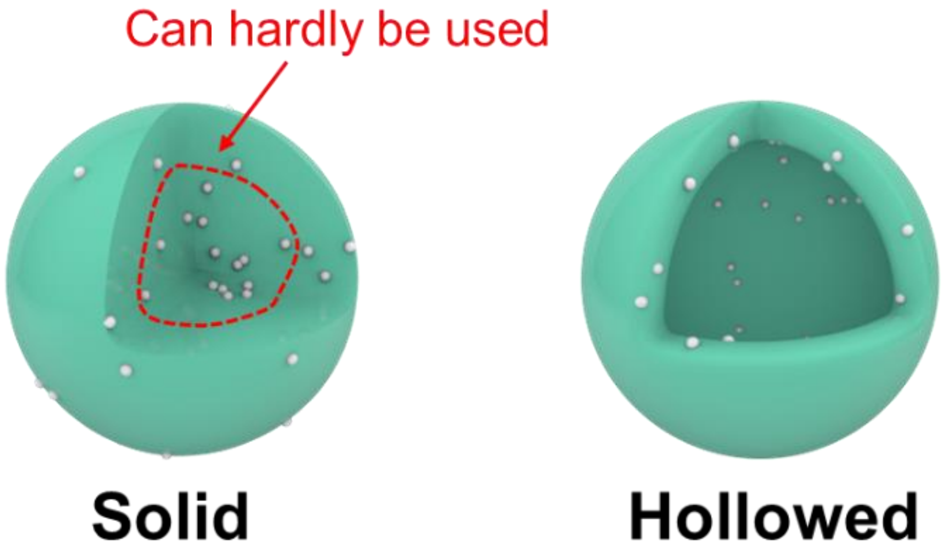

Figure S1. Schematic diagram of single atoms confined in solid and hollow carbon supports.

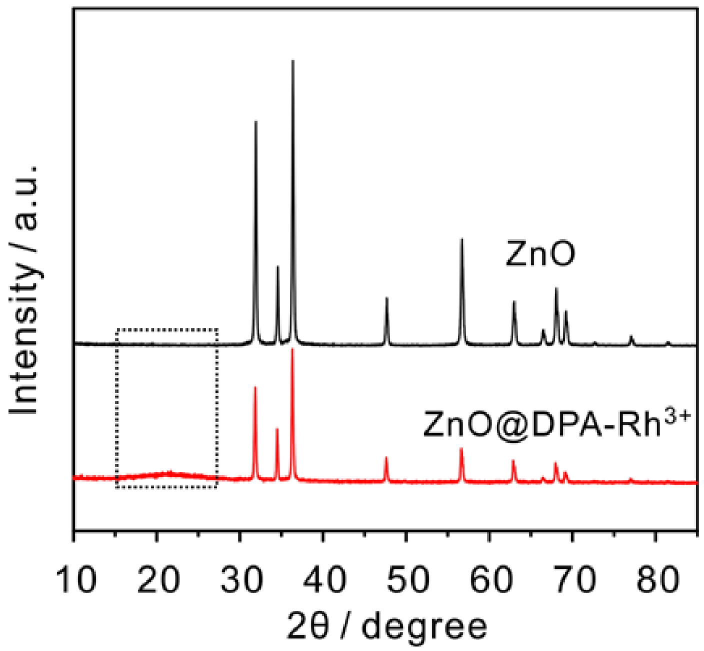

Figure S2. XRD patterns of ZnO nanorod and ZnO@ Rh^{3+}-DPA.

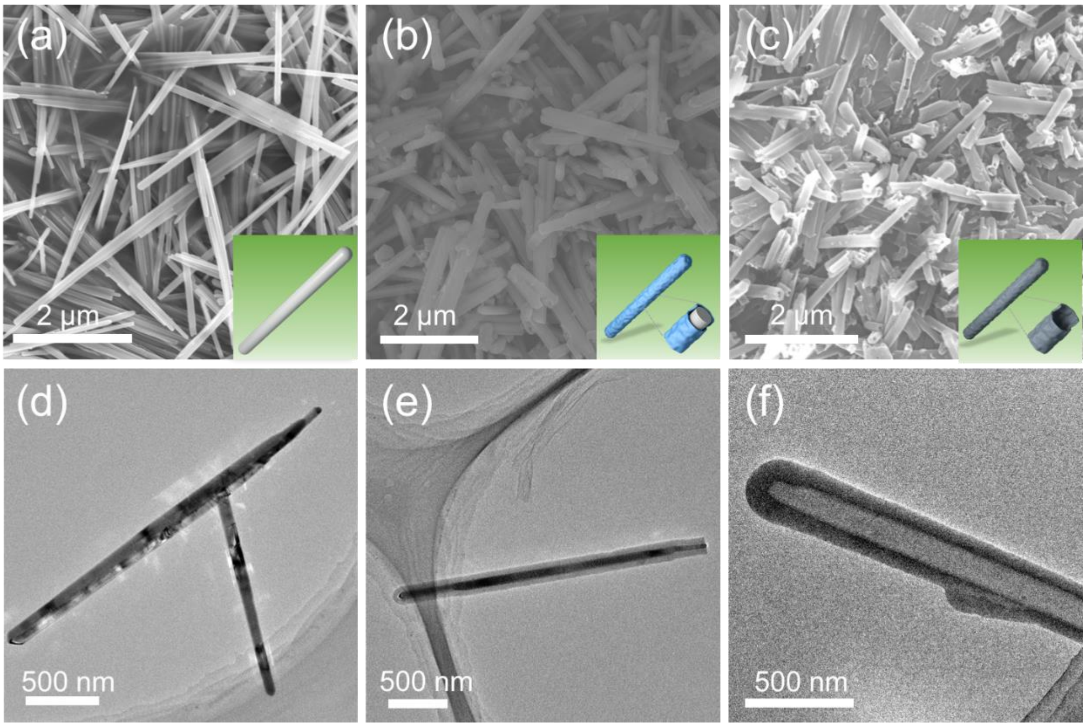

Figure S3. SEM and TEM images of ZnO nanorod (a, d), ZnO@ Rh^{3+}-DPA- (b, e) and Rh-SACs/HNCR (c, f). The inserts show the corresponding schematic illustrations.

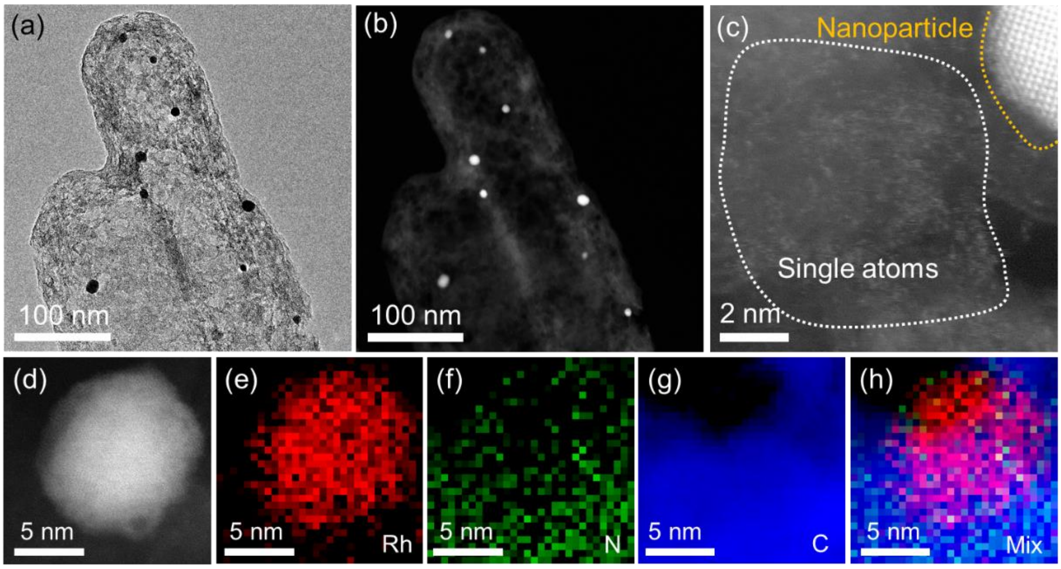

Figure S4. (a) TEM, (b) STEM images, (c) atomic-resolution STEM images of Rh-SACs/HNCR-1000. (d) HAADF STEM image and corresponding EELS maps of

(e) Rh, (f) N, (g) C and (h) composites.

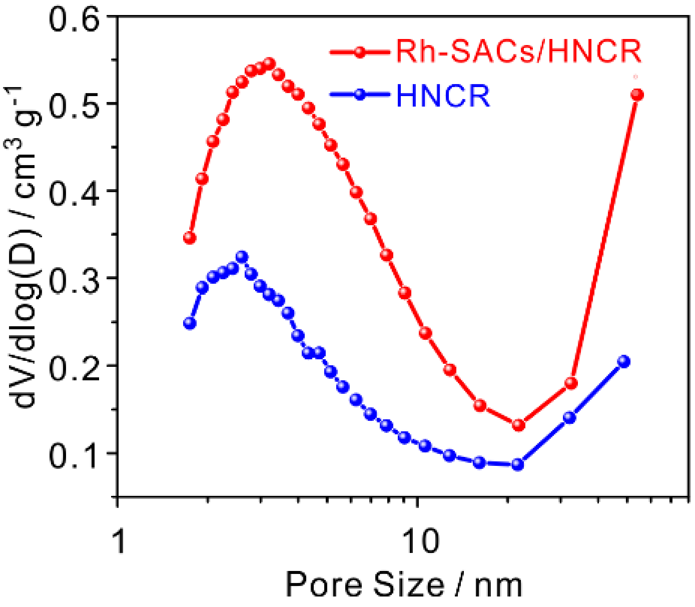

Figure S5. Pore size distribution of HNCR and Rh-SACs/HNCR.

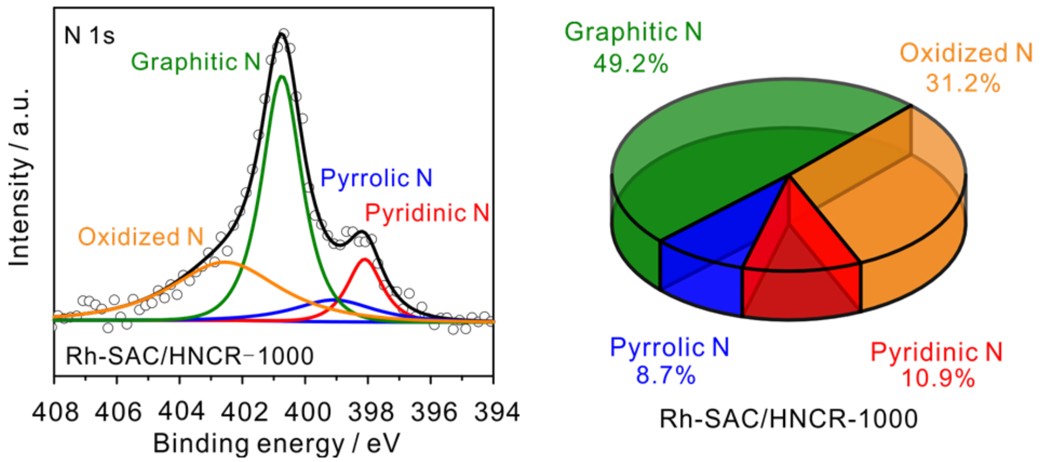

Figure S6. (a) The high-resolution N 1s XPS spectrum of Rh-SACs/HNCR-1000 and (b) corresponding percentage composition of N species.

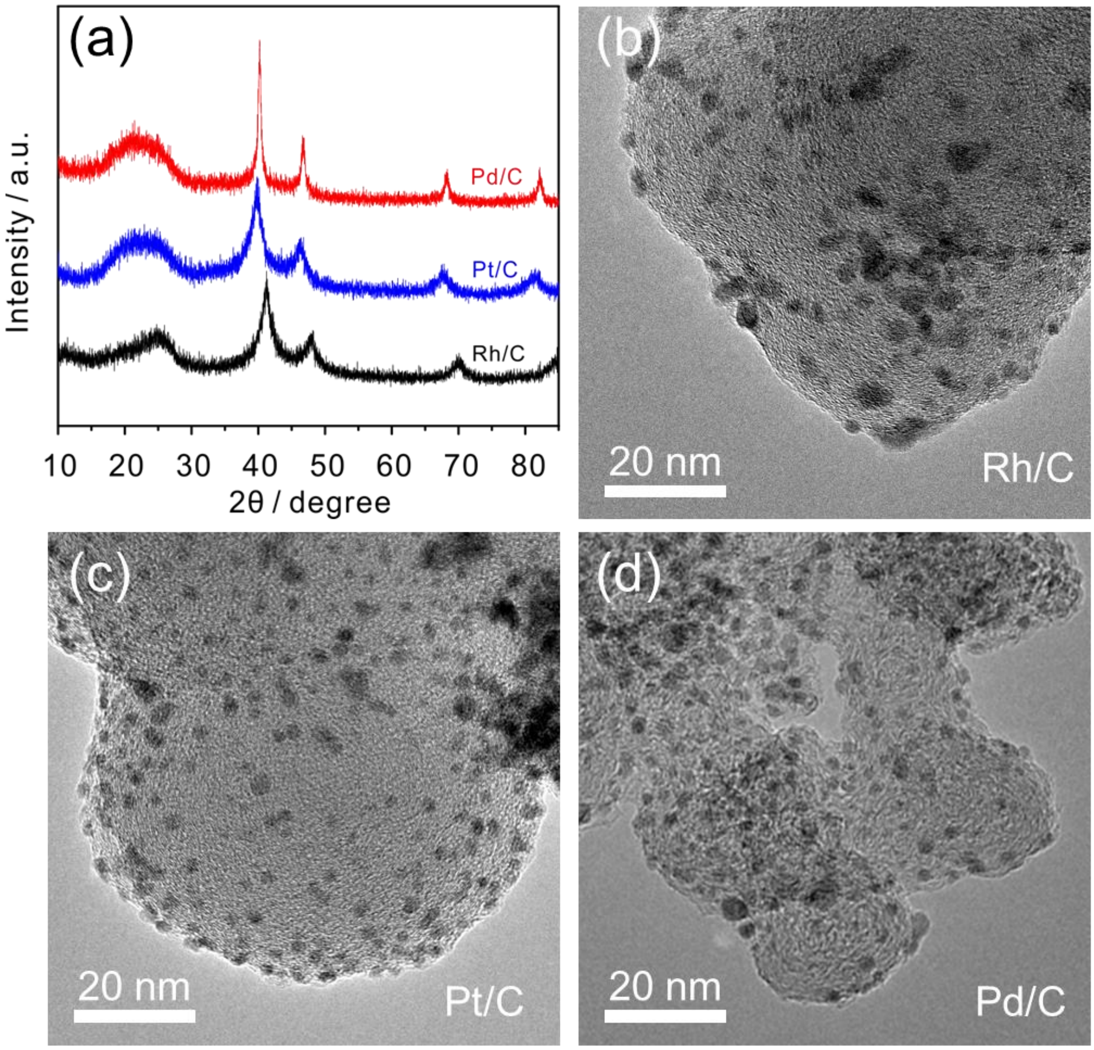

Figure S7. (a) XRD patterns of Rh/C, Pt/C and Pd/C. The TEM images of Rh/C (b), Pt/C (c) and Pd/C (d).

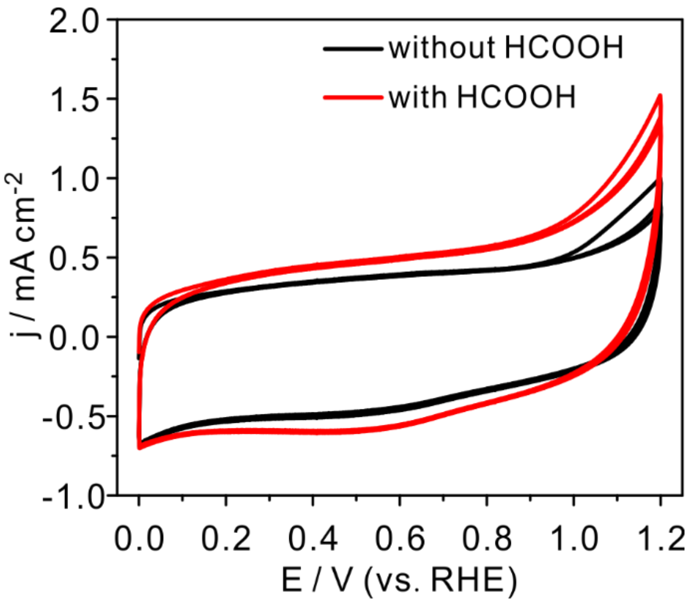

Figure S8. The CV curves of HNCR with and without the HCOOH in 0.5 M H_2SO_4.

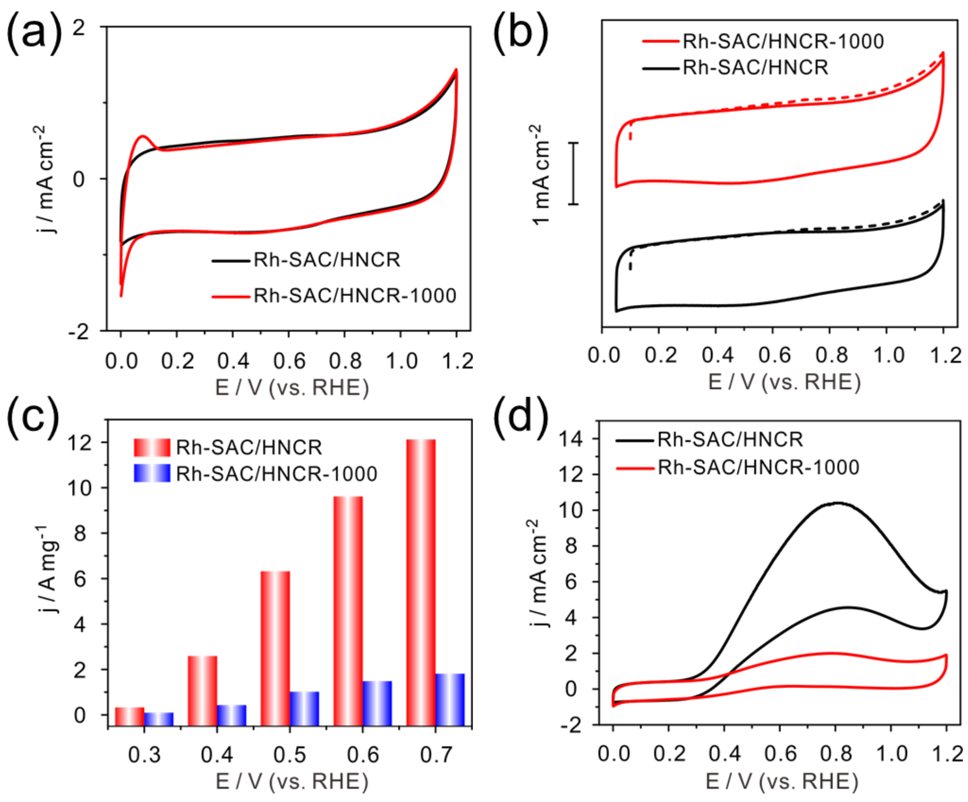

Figure S9. (a) CVs, (b) CO stripping curves and (c-d) normalized FAOR performances of Rh-SACs/HNCR and Rh-SACs/HNCR-1000.

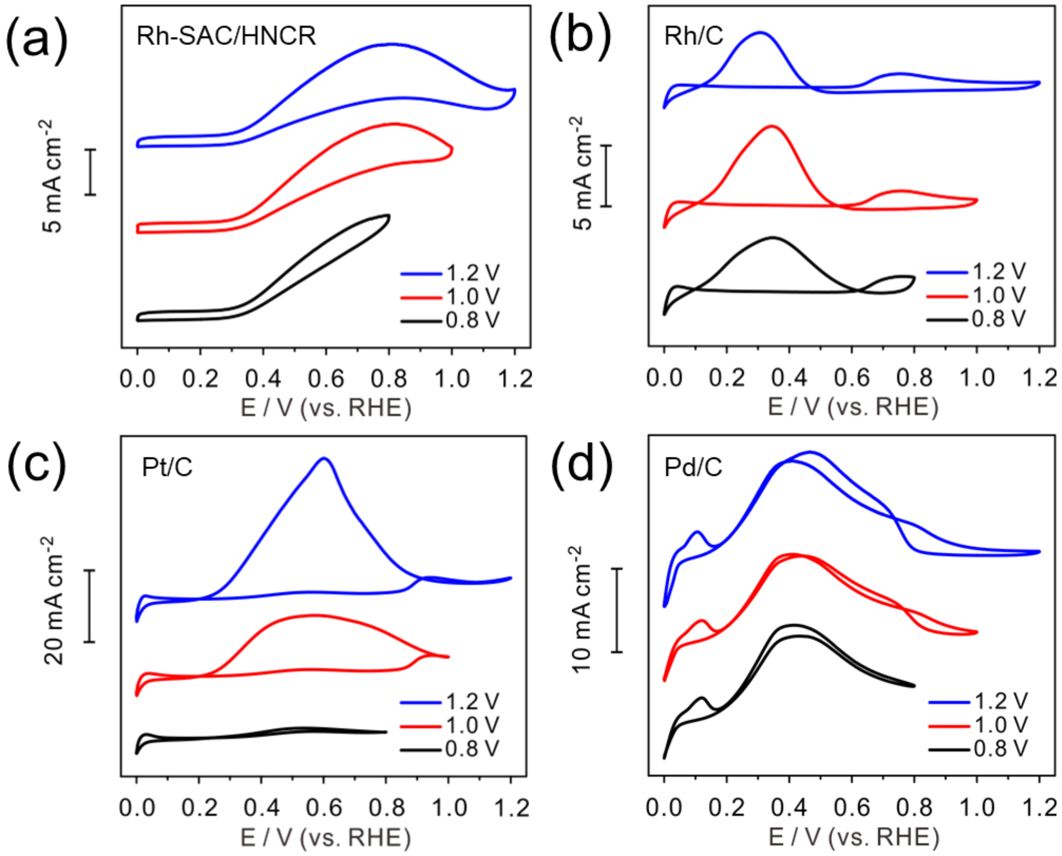

Figure S10. FAOR curves with different upper potential limit for (a) Rh-SACs/HNCR, (b) Rh/C, (c) Pt/C and (d) Pd/C.

Table S1. The comparison of total N contend and ratio of (pyridinic N + pyrrolic N)/ total N for Rh-SACs/HNCR and Rh-SACs/HNCR-1000.

Sample	Total N content	Ratio of (pyridinic N + pyrrolic N)/ total N
Rh-SACs/HNCR	2.65	0.379
Rh-SACs/HNCR-1000	1.62	0.196

Table S2 Comparison of FAOR performance of Rh-SACs/HNCR with previously reported values.

Samples	MA (A/mg)	electrolyte	ref
Rh-SACs/HNCR	13.1	0.5 M H_2SO_4+0.5 M HCOOH	**This work**
Rh/F-graphene-2	0.337	0.5 M H_2SO_4+0.5 M HCOOH	[1]
SA-Rh/CN	16.1	0.5 M H_2SO_4+0.5 M HCOOH	[2]
$Cu_{30}Pd_{70}$	1.193	0.1 M $HClO_4$ + 0.1 M HCOOH	[3]
PdBi nanodot	1.6285	0.5 M H_2SO_4+0.5 M HCOOH	[4]
Pt NWs	0.65	0.5 M H_2SO_4+0.5 M HCOOH	[5]
$Pt_{3.32}Cu$ HTBNF	0.78	0.25 M H_2SO_4+0.5 M HCOOH	[6]
Ir_1/CN	12.9	0.5 M H_2SO_4+0.5 M HCOOH	[7]
